# Propelling the paradigm shift from reductionism to systems nutrition

**DOI:** 10.1186/s12263-016-0549-8

**Published:** 2017-01-25

**Authors:** Jim Kaput, Giuditta Perozzi, Marijana Radonjic, Fabio Virgili

**Affiliations:** 1 Nestle Institute of Health Sciences, Lausanne, Switzerland; 2 CREA-NUT, Food & Nutrition Research Centre, Rome, Italy; 3 EdgeLeap, Utrecht, Netherlands

**Keywords:** Systems nutrition, Guidelines for biomedical research, Data standards, Women’s health

## Abstract

The complex physiology of living organisms represents a challenge for mechanistic understanding of the action of dietary bioactives in the human body and of their possible role in health and disease. Animal, cell, and microbial models have been extensively used to address questions that could not be pursued experimentally in humans, posing an additional level of complexity in translation of the results to healthy and diseased metabolism. The past few decades have witnessed a surge in development of increasingly sensitive molecular techniques and bioinformatic tools for storing, managing, and analyzing increasingly large datasets. Application of such powerful means to molecular nutrition research led to a major leap in study designs and experimental approaches yielding experimental data connecting dietary components to human health. Scientific journals bear major responsibilities in the advancement of science. As primary actors of dissemination to the scientific community, journals can impose rigid criteria for publishing only sound, reliable, and reproducible data. Journal policies are meant to guide potential authors to adopt the most updated standardization guidelines and shared best practices. Such policies evolve in parallel with the evolution of novel approaches and emerging challenges and therefore require constant updating. We highlight in this manuscript the major scientific issues that led to formulating new, updated journal policies for *Genes & Nutrition*, a journal which targets the growing field of nutritional systems biology interfacing personalized nutrition and preventive medicine, with the ultimate goal of promoting health and preventing or treating disease. We focus here on relevant issues requiring standardization in nutrition research. We also introduce new sections on human genetic variation and nutritional bioinformatics which follow the evolution of nutritional science into the twenty-first century.

## Introduction

The intensity and pace of modern science has accelerated at a breath-taking speed since the start of the modern genome era (circa 1990). High-throughput omics technologies have been applied to experimental designs and analytical methods based on reductionism and population averages, the “normal” scientific strategies [54] of the late twentieth and early twenty-first centuries. However, results produced under the current research model are unable to explain the complexity of biological processes. Nevertheless, the foundation of knowledge created during the post-genomic era justifies a “paradigm shift” [54] in how biomedical research needs to be conducted to understand how to maintain health and prevent or treat disease.

*Genes & Nutrition* (*G&N*) not only recognizes this scientific evolution but also plays a key role in promoting high-quality research in the trans-disciplinary fields linking nutrition, environment, genetics, and human health by setting high standards for studies accepted for publication. The tenth anniversary of *G&N* provided an opportunity not only to restate our mission but also to announce new guidelines for manuscripts submitted to this journal based on the significant advances in biomedical research in the past two decades. *Genes & Nutrition* was the recipient of many such advances and disseminated new ideas and results to the nutrition research community. Relevant examples are cited in the text below.

## Mission

*Genes & Nutrition* is an international, inter-disciplinary, peer-review journal for research on the relationship between genetics and nutrition, with the ultimate goal of providing knowledge for improving human health. Since its inception in 2006, *G&N* has seen a steady increase in readership, reflected in a credible impact factor that has recently resulted in its inclusion into the BioMed Central (BMC) family of free access journals.

In parallel with regular publications, the editorial team has stimulated readers’ involvement through editorials and commentaries, position papers (e.g., [46]), project ideas (e.g., [99]), literature highlights (e.g., [85]), as well as novel formulas leaving the authors a space for freely debating their opinions on the most “provocative” scientific issues relevant to the aims and scope of the journal (e.g., [75]). Our overall goal is to contribute to shaping the identity of the growing field of nutritional systems biology, interfacing personalized nutrition and preventive medicine, which started blooming within the molecular nutrition community in the twenty-first century.

## Guidelines, standards, and reproducibility of scientific data and findings

The application of advanced genomics technologies to nutrition (nutrigenomics) provides foundational knowledge for advancing nutrient–health associations for analysis of underlying mechanisms. High-throughput omics technologies are increasingly used to identify the often intertwined pathways of nutrient-dependent modulation of gene expression (at the gene, protein, metabolite levels) and/or epigenomic modifications, towards mechanistic understanding of nutrient-driven molecular processes at the system level [101]. Similar to all other fields of science applying such tools, successful outcome of sensitive molecular approaches requires a high degree of standardization at all levels of the experimental process, to limit confounders to a minimum and enable testing for reproducibility, thus avoiding generation of conflicting results. *Genes & Nutrition* has been frontline in requesting standardization of data, tools, and services. With this Editorial, we wish to endorse the agreed “Principles and Guidelines for Reporting Preclinical Research” (https://www.nih.gov/research-training/rigor-reproducibility/principles-guidelines-reporting-preclinical-research) jointly issued by major scientific journals to support scientific rigor and reproducibility [65]. In addition to standard requirements for human ethics and animal welfare, we highlight here the minimal requirements of our specific journal regarding standardization of nutritional studies, building and expanding on the shared policies of BMC Journals which request compliance to widely accepted guidelines (Table 1).

**Table 1:** Selected standards for biomedical research

Acronym	Name	Portal	Reference
BioDBcore	Core Attributes of Biological Databases	http://biocuration.org/communitystandards-biodbcore/	[35]
CIMR	Core Information for Metabolomics Reporting	http://www.metabolomics-msi.org/	
FAIR	Findable, Accessible, Interoperable, Reusable Data Principles	http://datafairport.org/fair-principles-living-document-menu	[107]
GCCP	Guidance on Good Cell Culture Practice	http://iclac.org/references/reading-guidelines/	[12, 25]
GSC	Genomics Standards Consortium	http://gensc.org/	[22]
ICLAC	International Cell Line Authentication Committee	http://iclac.org/wp-content/uploads/Advice-to-Scientists_09-Jan-2014.pdf	
MIABE	Minimum Information About a Bioactive Entity	http://www.psidev.info/node/394	[72]
MIAME	Minimum Information About a Microarray Experiment	https://biosharing.org/bsg-s000177	[5]
MIAPE	Minimum Information about a Proteomics Experiment	http://www.psidev.info/miape	
MIBBI	Minimum Information for Biological and Biomedical Investigations	https://biosharing.org/standards/?selected_facets=isMIBBI:true	[95]
IHM	International Human Microbiome Standards	http://www.microbiome-standards.org/	
MIGEN	Minimal Information about a Genotyping Experiment	http://migen.sourceforge.net/	[36]
MIQE	Minimum Information for Publication of Quantitative Real-Time PCR Experiment	http://miqe.gene-quantification.info/	[7]
MixS—MIGS/MIMS	Minimum Information about a (Meta)Genome Sequence	http://wiki.gensc.org/index.php?title=MIGS/MIMS	[27]
PGRCR	Principles and Guidelines for Reporting Preclinical Research	https://www.nih.gov/research-training/rigor-reproducibility/principles-guidelines-reporting-preclinical-research	
Stem Cells	Guidelines for Stem Cell Research and Clinical Translation	http://www.isscr.org/docs/default-source/guidelines/isscr-guidelines-for-stem-cell-research-and-clinical-translation.pdf?sfvrsn=2	
Women’s Health	Analysis of menstrual cycle phase		[1]

### Human genetic data

*Genes & Nutrition* was among the first specialty journals to focus on studies of the effects of nutrients on the expression of genetic information in humans and how genetic makeup alters the metabolism of nutrients [73, 83, 91, 108]. Perhaps no area of modern biomedical research has progressed as rapidly and contributed as profoundly as the analysis of genome sequence and structure. The NCBI list of completely or nearly completed sequenced genomes shows 3716 eukaryotes (plant and animal), 75,302 prokaryotes, 5962 viruses, 7799 plasmids, and 8748 organelle DNAs (http://www.ncbi.nlm.nih.gov/genome/browse/, accessed 16 October 2016).

The production of sequence data has far outpaced the ability to understand how genetic makeup influences phenotype or responds to nutrition and other environmental factors. Linking genetic loci that co-segregate with the trait within families (i.e., linkage analysis) proved highly successful using DNA molecular markers (rev in [74]). Over 1000 human monogenic diseases were identified by the year 2000 [42]. Exome and whole-genome sequencing (WGS) can now identify rare deleterious mutations in individuals [20], and comparison to parents or siblings enriches the chances of linking phenotype to genotype [69]. Although called monogenic disease, the age of disease onset, its severity, and time to outcome all differ between individuals carrying the same mutation demonstrating that other genes play a role disease etiology (e.g., [15]): in reality, no phenotype is produced by a single gene, except for those that are embryonically lethal. Exome and WGS have produced an additional surprise: any person’s genome typically contains ~100 genuine loss-of-function variants generating about 20 completely inactivated genes [60]. Such mutations may be phenotypically silent in the heterozygote: the Exome Aggregation Consortium (ExAC) analyzed 60,706 human exomes and discovered each genome contains ~54 variants reported as disease-causing [58] with ~41 occurring at >1% frequency in populations. In addition, this consortium found that 163 of 192 variants classified as pathogenic were benign or probably benign.

Candidate gene and genome-wide association studies (GWAS) utilize the same molecular technology as linkage studies to identify single nucleotide variants (SNVs) associated with complex diseases using a population-based as opposed to a family-based design. Thousands of GWA studies have been published associating one or more single nucleotide variants to ~800 phenotypes that include disease [106], diet intake [94], metabolism [8, 81], anthropometry [106], pharmacogenomics [17, 70], and brain-related disorders [56]. Over 15,000 trait–SNP associations at *p* value ≤5.0 × 10^−8^ have been amassed as of 2013 ([106] and https://www.ebi.ac.uk/gwas/). The majority of these SNP–trait associations have small effect sizes, and the sum of all variants identified for a trait explains only a small proportion of the phenotype [111].

Explaining the “missing or phantom heritability” has forced a re-evaluation of the assumptions and strategies of GWAS designs [26]. Table 2 describes some of the factors that influence genetic associations with phenotypes such as disease or response to diet. We recognize that few, if any, human research studies can incorporate all of these variables. However, interpretations of results must be tempered when relying on analysis of single omic or independent datasets.

**Table 2:** Missing heritability and the limitations of genome wide and candidate gene association studies

Limitation	Comments	References
Epistatic Interactions	Association studies analyze a single variable (e.g., SNP) with a trait. GWAS correct each SNP for multiple comparisons. Well documented in animal models with increasing numbers of examples in humans. Accounting for interactions decreased the amount of missing heritability. New analytical methods are being developed to test for interactions.	[11, 61, 62, 67, 105, 114]
Ascertainment bias	Many phenotypes such as type 2 diabetes or obesity were poorly characterized. For example, analysis of NHANES data demonstrated that body mass index was poorly associated with markers of cardiometabolic health. Not limited to GWAS	[97]
Gene–environment interactions	All organisms have genetic variation, producing phenotypic variation in response to environmental factors—this is the basis of natural selection. High-density genotyping, exome, and whole-genome sequencing have proved that each genome differs from all others. Adaptation to local environments has produced selection of gene variants—e.g., lactase persistence in Europe, Africa, and part of the Mideast and selection for metabolizing high-fat diets in Greenland Inuits. Experimental systems have demonstrated gene–diet interactions but as with SNP–disease studies, the effect size is small.	[24, 38, 39, 57, 71, 109, 113]
Epigenetics	An *epigenetic trait* is a stably heritable phenotype resulting from changes in a chromosome without alterations in the DNA sequence. Although not measured in most association studies, DNA methylation and chromatin modifications alter the expression of genetic information differentially in each tissue. Parent-of-origin genomic imprinting also alters gene regulation.	[14, 29, 88, 112]

*G&N* receives many manuscripts that describe statistically significant associations of a single nucleotide variable with a complex trait, such as obesity or response to a nutrient or diet. In almost all cases, the effect size is not reported and the studies are done in one population. In view of the limitations of SNV association studies (which are similar to those of GWAS), *G&N* will publish manuscripts associating one or two SNPs in one to several genes only when the effect size is reasonably high and conducted in more than one population. We encourage the use of classical statistical analysis of group differences when appropriate (e.g., by sex, age) but also systems approaches to both experimental designs and methods for data analysis.

### Designing human population/cohort studies

Risk factors calculated from population studies, most simply derived by comparing some measureable phenotype between control versus case (or intervention), are the average risk for the population (specifically, population attributable risk (PAR) [82]). Using an example from genetics, the PAR means that the incidence of a given disease or phenotype would change if the SNP or allele was eliminated in the population. Hence, PARs should not be considered as “personal risk factors.” Unfortunately, this important distinction is frequently disregarded.

Given the limitations of randomized clinical trials (RCTs), case intervention, and cohort studies for nutrition research (see [3, 32]), new experimental designs are needed to develop individual risk or benefit factors based on predictor variables from human clinical studies. *N*-of-1 studies are emerging as an experimental approach that first characterizes and then sorts individuals with similar metabolic profiles (e.g., [28, 44, 87]). While characterization and clustering can be done with baseline data, the response to acute challenges (e.g., mixed meal [92]) or short-term (e.g., weeks) interventions may be more informative given the variability in homeostasis between individuals [109]. Each individual serves as her/his own control, which eliminates ascertainment bias. This approach was first used in psychology studies [90] and then applied to clinical research [30, 31]. A trivial example is to compare individual male versus female or groups of males and females, although discovery-based algorithms (e.g., machine learning) may identify metabolic clusters based on all available data without a priori grouping. A number of *n*-of-1 studies have been published, including proposals to better define exactly how these studies should be conducted [10]. Since these concepts and approaches are not well tested, *G&N* will consider publishing results of studies analyzing individuals or groups identified by various clustering methods as long as the design can be justified and the results robust.

### Women’s health research

The US government passed the National Institute of Health Revitalization Act in 1993 (http://orwh.od.nih.gov/about/pdf/NIH-Revitalization-Act-1993.pdf) that required women and minorities to be included in federally funded clinical studies, with exceptions only when justified. Women had been largely excluded from many health research studies as a protective measure against unintended harm to the individual and her fetus [64]. Significant differences have been measured in a large number of biochemical and physiological systems that are consistent with sex-specific genetic profiles [77, 110]. These dissimilarities are in addition to the metabolic changes caused by exposure to hormones during the menstrual cycle and changes in hormone levels during pregnancy and lactation. Circulating levels of progesterone and estrogen are lowest during the early follicular phase when differences between a male and a female are probably least affected by hormone levels [1]. The result of sexual dimorphisms in metabolite levels [66] particularly in response to hormonal variations may lead to biased nutritional recommendations for men and women of all age groups.

While a review of sexual dimorphic differences between sexes is beyond the scope of this editorial, we also highlight the increasing awareness that fluctuating hormone levels during the menstrual cycle alter, for example, macronutrient metabolism [13], metabolic profiles [104], cardiometabolic markers [86], lipid kinetics [84], and the immune cell repertoire [50]. A recent review (of 146 articles winnowed by specific criteria from 1809) found a lack of consistency in determining the menstrual phase between studies and recommended the use of one of the six methods to assess cycle phase [1]. Recognizing that many existing systems nutrition studies will not have considered the menstrual cycle in comparing physiological and metabolic differences between males and females, new studies that adopt one of these methods to assess menstrual cycle phase will improve interpretation and reproducibility of results.

### Animal genetics

Many research studies in nutrition and toxicology relied on outbred mice to better reflect the structure of the population and translatability to humans (e.g., [37]). The use of outbred animals makes it difficult to appropriately power the experiment. In addition, maintaining a truly outbred population in breeding facilities is a significant challenge.

A distinct advantage of laboratory animals is the ability to develop inbred strains of defined genetic makeup. Mouse fanciers in Asia bred mice for coat color at least since the 1700s (https://www.genome.gov/10005832/background-on-the-history-of-the-mouse/). Researchers began using mice to study Mendelian inheritance patterns in the early 1900s when they began inbreeding mice first for coat color and subsequently for susceptibility to disease [103]. The limitation of using inbred animals is that each strain is genetically unique, often producing measurably different metabolic, developmental, or behavioral phenotypes. The Jackson Laboratory maintains not only a resource for mouse genetic data (http://www.informatics.jax.org/) but also phenotypic data based on its own in-house and published research results (http://phenome.jax.org/). Genetic variation can be incorporated into experiments by using several parental inbred strains and comparing physiological or transcriptomic responses.

A more recent development in analyzing complex traits was the creation of hybrid mouse diversity panel (HMDP) [59]. The HMDP consists of about 100 inbred strains generated from 30 parental inbred strains (e.g., BALB/c, C57BL/6) plus ~70 recombinant inbred mice (e.g., C57BL/6 × DBA). The combination of inbreeding these strains is to provide high-resolution mapping of genetic loci. DNA from many of these strains has been sequenced and all have been densely genotyped. The HMDP strains have substantial variation in metabolic, phenotypic traits, and disease susceptibilities with differential responses to changes in diet (e.g., [59]) which will allow for identifying interacting genes and gene–environment interactions. Data from studies involving the HMDP and some complementary human data can be accessed at the systems genetics resource [98]. Given the variability in phenotypes based on genetics, *G&N* will require strain designations and their commercial source for all studies.

### Animal diets

The complexity of diet compositions is widely acknowledged and yet the use of “standard chow diets” for nutritional studies in laboratory animals is common. Different lots of chow diets vary in chemical composition with the best-known examples being fatty acid composition [55] and estrogenic isoflavones [6, 18]. Mice fed different lots of chow have significantly different patterns of gene expression [53] confounding the ability to replicate gene–diet as well as microbiome studies. Ricci and Ulman (principals of Research Diets, Inc, New Brunswick, NJ) have developed what should be a simple meme for writing descriptions of animal diets [80]:

- Can I report it (can I tell others exactly what my animals were fed)?
- Can I repeat it (is there diet variability and will I be able to get the same results next year)?
- Can I revise it (as my hypotheses change, can I easily change the dietary components while keeping it otherwise matched to previous diets)?

The American Institute of Nutrition created the semi-purified AIN93A diet in 1993 [79] with revisions for growth, pregnancy, and lactation (AIN93G) and adult maintenance (AIN93M) in 1997 [78]. Semi-purified refers to the use of defined sources of carbohydrate, vitamins, minerals, protein source, and other essential nutrients plus dietary lipids in the form of corn, coconut, soy, or other plant-based oils (e.g., [45]). The chemical composition of an oil is also likely to vary depending upon the source and lot. The BIOCLAIMS project (http://bioclaims.uib.eu/) published a modified AIN93 diet in *Genes & Nutrition* that improved the essential fatty acid composition, polyunsaturated-to-saturated-fat ratio (>2), and use of oils without polyphenols or carotenes [34]. While these semi-purified component diets are standardized, chemical manipulations can be accommodated if nutrient-to-calorie ratios are maintained [80].

In line with the effort of ensuring reproducibility and scientific rigor, *G&N* requests authors to provide the full composition not only of experimental diets but also of the control standard diet employed in intervention studies on animal models. When sourced from commercial entities, the diet composition should be accompanied by the company name and diet identifier.

### Peripheral blood mononuclear cell (PMBC) analysis

Analyzing gene–nutrient interactions in humans is challenging because most human tissues cannot be sampled. In contrast, peripheral blood mononuclear cells (PBMCs) are easily obtained and pose no ethical barrier for most ages and conditions. The accessibility of these primary cells for studies of transcriptomic and DNA methylation analysis in response to diet and other environmental factors is highly tempting. PBMCs, however, are a highly diverse ecosystem. The mouse Immunological Genome Project (ImmGen) estimates that blood contains more than 250 types of cells and similar diversity likely exists in humans (https://www.immgen.org/). The number of these different PBMC types varies depending on the immunological status of the animal which would be defined by host and microbiome genome interactions [16] and their combined interactions with environmental factors. Although this consortium has yet to analyze RNA or DNA methylation levels in response to diet, they reported that 22% of PBMC transcripts differed by more than twofold across 39 inbred mouse, reflecting the influence of genetic background on gene expression.

Isolation procedures for distinct subsets of PBMC have been described (e.g., [33]) and methods have been developed to deconvolute PBMC expression [4] and DNA methylation [51] profiles to (albeit large) functional subgroups of cells. Arguments that transcriptomic or methylation profiles can be used as markers of disease or response to diet or other environmental influences are subject to the same criticism. Hence, *G&N* will only publish PBMC transcriptomics or DNA methylation studies with (semi-)purified cells or analyzed by methods that correlate experimental data with typical complete blood count (CBC).

### Cell line authentication

Scientific progress in biomedical (including nutrition) research was immensely boosted several decades ago by the introduction of in vitro cell models. Cell lines of mammalian origin are now available from almost all tissues, but genotypic and phenotypic changes have been described to occur in most of them over time (passage). Different laboratories may have no or different quality control procedures and hence the same cell lines may differ significantly [23]. The authentication and purity of cell lines are often undervalued by many researchers, who are frequently not aware of specific standards and guidelines ([9] and http://iclac.org/databases/cross-contaminations/). Major cell repositories should be the first to carry out unique identification of deposited cell lines, and the source of a cell line should always accompany the name identifier in published papers [102]. Alignment to good cell culture practices [12] published by the International Cell Line Authentication Committee (http://iclac.org/references/reading-guidelines/) is a prerequisite read for successful use of mammalian cell models in all branches of biomedical research. Funding agencies and scientific journals are increasingly requesting authors for proofs of cell line authentication ([21]). Adherence to standardized terminology in the naming of cell lines is explicitly requested to journals and consequently to the authors [23].

A high proportion of manuscripts received by *Genes & Nutrition* employ stable cell lines to investigate nutrient-dependent modulation of metabolic pathways in different tissues. To ensure reproducibility and reliability of research results, and in line with the policies adopted by major scientific journals (NIH Rigor and Reproducibility: Principles and Guidelines for Reporting Preclinical Research and Endorsement by major journals - https://www.nih.gov/research-training/rigor-reproducibility/principles-guidelines-reporting-preclinical-research), manuscripts submitted to *G&N* should report the source, authentication, and contamination testing in the “Materials and Methods” section, performed according to widely accepted international guidelines ([2] and references therein). Studies involving human embryonic stem cells must meet not only these exacting research standards but also ethical and legal standards applicable to all human research experiments. An international compilation of human research standards is available from the Office of Human Research Protections of the US Department of Health and Human Services (https://www.hhs.gov/ohrp/).

### Microbiome

The use of high-throughput sequence technologies spurred the study of the microbiomes that live in and on humans and other organisms. Many investigators have studied the gut microbial community because of the obvious involvement in gastrointestinal disorders such as inflammatory bowel disease [52]. The microbial ecosystem, however, plays a key role in metabolizing and producing nutrients (e.g., [63]), modulating the immune system [96], and producing signaling molecules affecting the gut–brain axis [40]. A primer for researchers for conducting a microbiome study has recently been published [27] and refinements in standards are likely to be developed as this field matures.

### Natural bioactives

Disease prevention and treatment using natural products and their analogs has a long and rich history in Eastern and Western medicine. The quest for and application of bioactive extracts and purified chemical substances of nutritional interest from both herbal and animal origin is attracting increasing attention among clinical and basic science investigators (e.g., [19, 93]).

An expanding focus of this research is aimed at elucidating the mechanism of action of “natural compounds” and the presumed consequences for metabolic health. Segments of the non-scientific community, and in particular the media, avidly follow the developments of this research field. The phenomenological/ethno-medical field has contributed observational and anecdotal results stimulating researchers to propose robust, science-based mechanisms of action. In particular, the definition of key molecular pathways affected by phytochemical and nutritional bioactive compounds is of crucial importance to understand health effects. Unfortunately, research in this area often lacked rigor, reproducibility, and molecular detail, and its outcomes were therefore received with justifiable skepticism.

*G&N* publishes original research papers and reviews dealing with bioactive constituents of traditional or novel foods, and of botanical extracts targeted at promoting health and preventing or treating disease. According to our scopes and aims, *G&N* encourages studies of the effects of bioactives on the modulation of biochemical pathways, cellular signaling, and gene expression.

In line with our quest for standardization, studies dealing with the effects of natural compounds or food/botanical extracts should report a complete characterization and standardization of the compound/s under study, to allow reproducibility. Exceptions to this guideline may be possible with very strong evidence of the importance of their results or when dealing with novel botanical extracts of extreme interest, whose effects have never been reported in the available literature, and for which chemical characterization is in progress.

## Nutritional bioinformatics

As in other life science disciplines, data science is an indispensable component of contemporary nutrition research. With growing possibilities to measure, store, and analyze data and knowledge (e.g., [100]), availability and means to effectively integrate and mine information is rapidly becoming a key determinant of successful translation of nutrition research into human health benefits (e.g., [41, 89]). Application of existing and development of new computational methods (e.g., [68]) are being used for systems analysis of high dimensional, multi-omic data sets (e.g., [47, 49]) and their integration with physiological and clinical endpoints to infer health effects of interventions [48].

To promote leveraging advances in data science to facilitate goals of nutrition research, *G&N* has established a novel “Nutritional Bioinformatics” section. Traditionally, the disciplines of bioinformatics and nutrition research have evolved independently. In this new section, we emphasize the connection between the two, recognizing the specific requirements for data science approaches in the context of nutrition research (e.g., dealing with subtle and broad/systems effects of dietary interventions) as well as establishing state-of-art data handling practices within classical nutritional studies. *G&N* actively encourages efforts towards development and application of data analytics methods and resources tailored to use in nutrition research. The “Nutritional Bioinformatics” section welcomes submission of articles addressing development and utilization of novel analytics approaches, software, tools, and data resources relevant in the context of nutrition research. Descriptions of newly developing research infrastructures aimed at providing services to the nutrition research community are also encouraged and welcomed. Compliance of submissions with standards of good data practices, such as FAIR guidelines (data is required to be Findable, Accessible, Interoperable, and Reusable–[107]) is essential.

## Summary

Reproducibility, or at least the ability to understand the reasons for different results and outcomes in preclinical and clinical research experiments, is now demanding the attention of researchers, funding agencies, and the public (e.g., [76]). The intent of this editorial was to provide guidance for improving the quality of systems nutrition research (Table 3). We recognize, however, that new developments will require constant updating of methods, experimental designs, and computational analysis. As our climate changes and population growth increases to the expected nine billion by 2050, our field of research must continue to produce high-quality, reliable results that can be translated into health-promoting action [43]. *Genes & Nutrition* remains committed to promoting and publishing this research in a timely fashion to improve nutrition knowledge for applications for personal and public health.

**Table 3:** Summary of genes and nutrition publication guidelines

1. Standardization/reproducibility of data and findings. Manuscripts submitted to *G&N* should contain the necessary information allowing evaluation of alignment with widely accepted best practices (see Table 2 for specific guidelines). These standards include descriptions of reference materials and reagents, study design, laboratory protocols, data analysis, and reporting. We recommend authors to refer to the MIBBI Portal (Minimum Information for Biological and Biomedical Investigations) for prescriptive checklists for reporting biological and biomedical research where applicable.
2. Gene variants. *G&N* manuscripts reporting associations between single SNPs and complex phenotypes or single SNPs and response to diet will not be sent for peer review unless (i) the effect size is large, (ii) *p* values significant and corrected for multiple comparison, and (iii) replicated in a second study. Complex phenotypes include diseases, anthropometric measures (e.g., body weight or body mass index), and intermediate risk factors (e.g., levels or changes in fasting blood parameters).
3. Women’s health research. Sexual dimorphism in metabolic response should be assessed, and when possible, the phase of the menstrual cycle phase analyzed by one of the six methods described in [1].
4. Animal genetics. *G&N* will require strain designations and their commercial source for all studies.
5. Animal diets. Different lots of chow diets vary in chemical composition with the best examples being fatty acid composition [55] and estrogenic isoflavones [6, 18]. Ricci and Ulman (principals of Research Diets, Inc, New Brunswick, NJ) have developed what should be a simple meme for writing descriptions of experimental diets [80]
6. Peripheral blood mononuclear cell (PMBC) analysis. The accessibility of PBMCs for studies of transcriptomic and DNA methylation analysis in response to diet and other environmental factors is highly tempting. PBMCs, however, are a highly diverse ecosystem. Isolation procedures for distinct subsets of PBMC have been described (e.g., [33]), and methods have been developed to deconvolute PBMC expression [4] and DNA methylation [51] profiles to (albeit large) functional subgroups of cells.
7. Cell line authentication. Different laboratories may have no or different quality control procedures and hence the “same” cell lines may differ significantly [23]. The authentication and purity of cell lines are often undervalued by many researchers, who are frequently not aware of specific standards and guidelines ([9] and http://iclac.org/databases/cross-contaminations/). Alignment to good cell culture practices [12] published by the International Cell Line Authentication Committee is a prerequisite read for successful use of mammalian cell models in all branches of biomedical research.
8. Microbiome. A primer for researchers for conducting a microbiome study has recently been published [27] and refinements in standards are likely to be developed as this field matures.
9. Natural compounds. Studies dealing with the effect of natural compounds or food/botanical extracts should report characterization and standardization of the material utilized to allow reproducibility as a prerequisite for peer reviewing. Standard reporting methods are described in [72].
10. Data standards. Compliance of submissions with standards of good data practices, such as FAIR guidelines (data is required to be Findable, Accessible, Interoperable, and Reusable—[107]) is essential.
